# Supplementary material for: Study of the mental health status of medical personnel dealing with new coronavirus pneumonia
Source: PLoS One. 2020 May 19;15(5):e0233145. doi: 10.1371/journal.pone.0233145 (PMC7237008; doi:10.1371/journal.pone.0233145)
Supplement: S1 File — (DOC) [file pone.0233145.s001.doc]

**症状自评量表SCL-90**

编号 姓名 性别 年龄 岁 日期

指导语：下面有90条测验项目，列出了有些人可能有的问题，仔细阅读每一条，根据自己现在或最近一星期内的感觉，在相应的方格内划一个“√”。必须逐条填写不可遗漏，每一项只能划一个“√”，不能划两个或更多。自我评定的五个等级：

1. 无：自觉并无该项问题（症状）；
2. 轻度：自觉有该问题，但发生得并不频繁、不严重；
3. 中度：自觉有该项症状，其严重程度为轻到中度；
4. 偏重：自觉常有该项症状，其程度为中到严重；
5. 严重：自觉该症状的频度和强度都十分严重。

| 条目 | 无 | 轻度 | 中度 | 偏重 | 严重 |
| --- | --- | --- | --- | --- | --- |
| 1. 头痛 |  |  |  |  |  |
| 1. 神经过敏，心中不踏实 |  |  |  |  |  |
| 1. 头脑中有不必要的想法或字句盘旋 |  |  |  |  |  |
| 1. 头晕或晕倒 |  |  |  |  |  |
| 1. 对异性的兴趣减退 |  |  |  |  |  |
| 1. 对旁人责备求全 |  |  |  |  |  |
| 1. 感到别人能控制您的思想 |  |  |  |  |  |
| 1. 责怪别人制造麻烦 |  |  |  |  |  |
| 1. 忘性大 |  |  |  |  |  |
| 1. 担心自己的衣饰整齐及仪态的端正 |  |  |  |  |  |
| 1. 容易烦恼和激动 |  |  |  |  |  |
| 1. 胸痛 |  |  |  |  |  |
| 1. 害怕空旷的场所或街道 |  |  |  |  |  |
| 1. 感到自己的精力下降，活动减慢 |  |  |  |  |  |
| 1. 想结束自己的生命 |  |  |  |  |  |
| 1. 听到旁人听不到的声音 |  |  |  |  |  |
| 1. 发抖 |  |  |  |  |  |
| 1. 感到大多数人都不可信任 |  |  |  |  |  |
| 1. 胃口不好 |  |  |  |  |  |
| 1. 容易哭泣 |  |  |  |  |  |
| 1. 同异性相处时感到害羞不自在 |  |  |  |  |  |
| 1. 感到受骗，中了圈套或有人想抓住您 |  |  |  |  |  |
| 1. 无缘无故地突然感到害怕 |  |  |  |  |  |
| 1. 自己不能控制地大发脾气 |  |  |  |  |  |
| 1. 怕单独出门 |  |  |  |  |  |
| 1. 经常责怪自己 |  |  |  |  |  |
| 1. 腰痛 |  |  |  |  |  |
| 1. 感到难以完成任务 |  |  |  |  |  |
| 1. 感到孤独 |  |  |  |  |  |
| 1. 感到苦闷 |  |  |  |  |  |
| 1. 过分担忧 |  |  |  |  |  |
| 1. 对事物不感兴趣 |  |  |  |  |  |
| 1. 感到害怕 |  |  |  |  |  |
| 1. 您的感情容易受到伤害 |  |  |  |  |  |
| 1. 旁人能知道您的私下想法 |  |  |  |  |  |
| 1. 感到别人不理解您、不同情您 |  |  |  |  |  |
| 1. 感到人们对您不友好，不喜欢您 |  |  |  |  |  |
| 1. 做事必须做得很慢以保证做得正确 |  |  |  |  |  |
| 1. 心跳得很厉害 |  |  |  |  |  |
| 1. 恶心或胃部不舒服 |  |  |  |  |  |
| 1. 感到比不上他人 |  |  |  |  |  |
| 1. 肌肉酸痛 |  |  |  |  |  |
| 1. 感到有人在监视您、谈论您 |  |  |  |  |  |
| 1. 难以入睡 |  |  |  |  |  |
| 1. 做事必须反复检查 |  |  |  |  |  |
| 1. 难以做出决定 |  |  |  |  |  |
| 1. 怕乘电车、公共汽车、地铁或火车 |  |  |  |  |  |
| 1. 呼吸有困难 |  |  |  |  |  |
| 1. 一阵阵发冷或发热 |  |  |  |  |  |
| 1. 因为感到害怕而避开某些东西、场合或活动 |  |  |  |  |  |
| 1. 脑子变空了 |  |  |  |  |  |
| 1. 身体发麻或刺痛 |  |  |  |  |  |
| 1. 喉咙有梗塞感 |  |  |  |  |  |
| 1. 感到前途没有希望 |  |  |  |  |  |
| 1. 不能集中注意力 |  |  |  |  |  |
| 1. 感到身体的某一部分软弱无力 |  |  |  |  |  |
| 1. 感到紧张或容易紧张 |  |  |  |  |  |
| 1. 感到手或脚发重 |  |  |  |  |  |
| 1. 想到死亡的事 |  |  |  |  |  |
| 1. 吃得太多 |  |  |  |  |  |
| 1. 当别人看着您或谈论您时感到不自在 |  |  |  |  |  |
| 1. 有一些不属于您自己的想法 |  |  |  |  |  |
| 1. 有想打人或伤害他人的冲动 |  |  |  |  |  |
| 1. 醒得太早 |  |  |  |  |  |
| 1. 必须反复洗手、点数或触摸某些东西 |  |  |  |  |  |
| 1. 睡得不稳不深 |  |  |  |  |  |
| 1. 有想摔坏或破坏东西的想法 |  |  |  |  |  |
| 1. 有一些别人没有的想法 |  |  |  |  |  |
| 1. 感到对别人神经过敏 |  |  |  |  |  |
| 1. 在商店或电影院等人多的地方感到不自在 |  |  |  |  |  |
| 1. 感到任何事情都很困难 |  |  |  |  |  |
| 1. 一阵阵恐惧或惊恐 |  |  |  |  |  |
| 1. 感到公共场合吃东西很不舒服 |  |  |  |  |  |
| 1. 经常与人争论 |  |  |  |  |  |
| 1. 单独一人时神经很紧张 |  |  |  |  |  |
| 1. 别人对您的成绩没有做出恰当的评价 |  |  |  |  |  |
| 1. 即使和别人在一起也感到孤单 |  |  |  |  |  |
| 1. 感到坐立不安，心神不定 |  |  |  |  |  |
| 1. 感到自己没有什么价值 |  |  |  |  |  |
| 1. 感到熟悉的东西变成陌生或不像是真的 |  |  |  |  |  |
| 1. 大叫或摔东西 |  |  |  |  |  |
| 1. 害怕会在公共场合晕倒 |  |  |  |  |  |
| 1. 感到别人想占您的便宜 |  |  |  |  |  |
| 1. 为一些有关“性”的想法而很苦恼 |  |  |  |  |  |
| 1. 您认为应该因为自己的过错而受到惩罚 |  |  |  |  |  |
| 1. 感到要很快把事情做完 |  |  |  |  |  |
| 1. 感到自己的身体有严重问题 |  |  |  |  |  |
| 1. 从未感到和其他人很亲近 |  |  |  |  |  |
| 1. 感到自己有罪 |  |  |  |  |  |
| 1. 感到自己的脑子有毛病 |  |  |  |  |  |

**测验目的：**

本测验的目的是从感觉、情感、思维、 意识、行为直到生活习惯、人际关系、饮食睡眠等多种角度，评定一个人是否有某种心理症状及其严重程度如何。它对有心理症状（即有可能处于心理障碍或心理障碍边缘）的人有良好的区分能力。适用于测查某人群中那些人可能有心理障碍、某人可能有何种心理障碍及其严重程度如何。不适合于躁狂症和精神分裂症。

**测验功能：**

SCL90 对有心理症状（即有可能处于心理障碍或心理障碍边缘）的人有良好的区分能力。适用于测查某人群中那些人可能有心理障碍、某人可能有何种心理障碍及其严重程度如何。 可用于临床上检查是否存在身心疾病，各大医院大都要使用本测验诊断患者的心理和精神问题。本测验不仅可以自我测查，也可以对他人（如其行为异常，有患精神或心理疾病的可能）进行核查，假如发现得分较高，则表明急需治疗。

**分析统计指标：**

（一）总分

1．总分是90个项目所得分之和。

2．总症状指数，也称总均分，是将总分除以90（＝总分÷90）。

3．阳性项目数是指评为1－4分的项目数，阳性症状痛苦水平是指总分除以阳性项目数（＝总分÷阳性项目数）。

4．阳性症状均分是指总分减去阴性项目（评为0的项目）总分，再除以阳性项目数。

（二）因子分

SCL－90包括9个因子，每一个因子反映出病人的某方面症状痛苦情况，通过因子分可了解症状分布特点。

因子分＝组成某一因子的各项目总分／组成某一因子的项目数

9个因子含义及所包含项目为：

1．躯体化：包括1，4，12，27，40，42，48，49，52，53，56，58共12项。该因子主要反映身体不适感，包括心血管、胃肠道、呼吸和其他系统的主诉不适，和头痛、背痛、肌肉酸痛，以及焦虑的其他躯体表现。

2．强迫症状：包括了3，9，10，28，38，45，46，51，55，65共10项。主要指那些明知没有必要，但又无法摆脱的无意义的思想、冲动和行为，还有一些比较一般的认知障碍的行为征象也在这一因子中反映。

3．人际关系敏感：包括6，21，34，36，37，41，61，69，73共9项。主要指某些个人不自在与自卑感，特别是与其他人相比较时更加突出。在人际交往中的自卑感，心神不安，明显不自在，以及人际交流中的自我意识，消极的期待亦是这方面症状的典型原因。

4．抑郁：包括5，14，15，20，22，26，29，30，31，32，54，71，79共13项。苦闷的情感与心境为代表性症状，还以生活兴趣的减退，动力缺乏，活力丧失等为特征。还反映失望，悲观以及与抑郁相联系的认知和躯体方面的感受，另外，还包括有关死亡的思想和自杀观念。

5．焦虑：包括2，17，23，33，39，57，72，78，80，86共10项。一般指那些烦躁，坐立不安，神经过敏，紧张以及由此产生的躯体征象，如震颤等。测定游离不定的焦虑及惊恐发作是本因子的主要内容，还包括一项解体感受的项目。

6．敌对：包括11，24，63，67，74，81共6项。主要从三方面来反映敌对的表现：思想、感情及行为。其项目包括厌烦的感觉，摔物，争论直到不可控制的脾气暴发等各方面。

7．恐怖：包括13，25，47，50，70，75，82共7项。恐惧的对象包括出门旅行，空旷场地，人群或公共场所和交通工具。此外，还有反映社交恐怖的一些项目。

8．偏执：包括8，18，43，68，76，83共6项。本因子是围练偏执性思维的基本特征而制订：主要指投射性思维，敌对，猜疑，关系观念，妄想，被动体验和夸大等。

9．精神病性：包括7，16，35，62，77，84，85，87，88，90共10项。反映各式各样的急性症状和行为，限定不严的精神病性过程的指征。此外，也可以反映精神病性行为的继发征兆和分裂性生活方式的指征。

此外还有19，44，59，60，64，66，89共7个项目未归入任何因子，反映睡眠及饮食情况，分析时将这7项作为附加项目或其他，作为第10个因子来处理，以便使各因子分之和等于总分。

各因子的因子分的计算方法是：各因子所有项目的分数之和除以因子项目数。例如强迫症状因子各项目的分数之和假设为30，共有10个项目，所以因子分为3。在1—5评分制中，粗略简单的判断方法是看因子分是否超过3分，若超过3分，即表明该因子的症状已达到中等以上严重程度。下面是正常成人SCL－90的因子分常模，如果因子分超过常模即为异常。

**计分方法：表1—2：SCL—90测验结果处理**

| 因子 | 因子含义 | 项 目 | T分=项目总分/项目数 | T分 |
| --- | --- | --- | --- | --- |
| F1 | 躯体化 | 1、4、12、27、40、42、48、49、52、53、56、58 | /12 |  |
| F2 | 强迫 | 3、9、10、28、38、45、46、51、55、65 | /10 |  |
| F3 | 人际关系 | 6、21、34、36、37、41、61、69、73 | /9 |  |
| F4 | 抑郁 | 5、14、15、20、22、26、29、30、31、32、54、71、79 | /13 |  |
| F5 | 焦虑 | 2、17、23、33、39、57、72、78、80、86 | /10 |  |
| F6 | 敌对性 | 11、24、63、67、74、81 | /6 |  |
| F7 | 恐怖 | 13、25、47、50、70、75、82 | /7 |  |
| F8 | 偏执 | 8、18、43、68、76、83 | /6 |  |
| F9 | 精神病性 | 7、16、35、62、77、84、85、87、88、90 | /10 |  |
| F10 | 睡眠及饮食 | 13、25、47、50、70、75、82 | /7 |  |

**正常成人SCL—90的因子分常模**

| 项 目 | M ±SD | 项 目 | M±SD |
| --- | --- | --- | --- |
| 躯体化 | 1.37±0.48 | 敌对性 | 1.46±0.55 |
| 强迫 | 1.62±0.58 | 恐怖 | 1.23±0.41 |
| 人际关系 | 1.65±0.61 | 偏执 | 1.43±0.57 |
| 抑郁 | 1.50±0.59 | 精神病性 | 1.29±0.42 |
| 焦虑 | 1.39±0.43 |  |  |

**得分解释：**

总症状指数

是指总的来看，被试的自我症状评价介于“没有”到“严重”的哪一个水平。总症状指数的分数在0～0.5之间，表明被试自我感觉没有量表中所列的症状；在0.5～1.5之间，表明被试感觉有点症状，但发生得并不频繁；在1.5～2.5之间，表明被试感觉有症状，其严重程度为轻到中度；在2.5～3.5之间，表明被试感觉有症状，其程度为中到严重；在3.5～4之间表明被试感觉有，且症状的频度和强度都十分严重。

阳性项目数

是指被评为1-4分的项目数分别是多少，它表示被试在多少项目中感到“有症状”。

阴性项目数

是指被评为0分的项目数，它表示被试“无症状”的项目有多少。

阳性症状均分

是指个体自我感觉不佳的项目的程度究竟处于哪个水平。其意义与总症状指数的相同。

因子分

SCL-90包括9个因子，每一个因子反映出个体某方面的症状情况，通过因子分可了解症状分布特点。当个体在某一因子的得分大于2时，即超出正常均分，则个体在该方面就很有可能有心理健康方面的问题。

⑴．躯体化

主要反映身体不适感，包括心血管、胃肠道、呼吸和其他系统的不适，和头痛、背痛、肌肉酸痛，以及焦虑等躯体不适表现。

该分量表的得分在0-48分之间。得分在24分以上，表明个体在身体上有较明显的不适感，并常伴有头痛、肌肉酸痛等症状。得分在12分以下，躯体症状表现不明显。总的说来，得分越高，躯体的不适感越强；得分越低，症状体验越不明显。

⑵．强迫症状

主要指那些明知没有必要，但又无法摆脱的无意义的思想、冲动和行为，还有一些比较一般的认知障碍的行为征象也在这一因子中反映。

该分量表的得分在0-40分之间。得分在20分以上，强迫症状较明显。得分在10分以下，强迫症状不明显。总的说来，得分越高，表明个体越无法摆脱一些无意义的行为、思想和冲动，并可能表现出一些认知障碍的行为征兆。得分越低，表明个体在此种症状上表现越不明显，没有出现强迫行为。

⑶．人际关系敏感

主要是指某些人际的不自在与自卑感，特别是与其他人相比较时更加突出。在人际交往中的自卑感，心神不安，明显的不自在，以及人际交流中的不良自我暗示，消极的期待等是这方面症状的典型原因。 　　该分量表的得分在0-36分之间。得分在18分以上，表明个体人际关系较为敏感，人际交往中自卑感较强，并伴有行为症状（如坐立不安，退缩等）。得分在9分以下，表明个体在人际关系上较为正常。总的说来，得分越高，个体在人际交往中表现的问题就越多，自卑，自我中心越突出，并且已表现出消极的期待。得分越低，个体在人际关系上越能应付自如，人际交流自信、胸有成竹，并抱有积极的期待。

⑷．抑郁

苦闷的情感与心境为代表性症状，还以生活兴趣的减退，动力缺乏，活力丧失等为特征。还表现出失望、悲观以及与抑郁相联系的认知和躯体方面的感受，另外，还包括有关死亡的思想和自杀观念。

该分量表的得分在0-52分之间。得分在26分以上，表明个体的抑郁程度较强，生活缺乏足够的兴趣，缺乏运动活力，极端情况下，可能会有想死亡的思想和自杀的观念。得分在13分以下，表明个体抑郁程度较弱，生活态度乐观积极，充满活力，心境愉快。总的说来，得分越高，抑郁程度越明显，得分越低，抑郁程度越不明显。

⑸．焦虑

一般指那些烦躁，坐立不安，神经过敏，紧张以及由此产生的躯体征象，如震颤等。

该分量表的得分在0-40分之间。得分在20分以上，表明个体较易焦虑，易表现出烦躁、不安静和神经过敏，极端时可能导致惊恐发作。得分在10分以下，表明个体不易焦虑，易表现出安定的状态。总的说来，得分越高，焦虑表现越明显。得分越低，越不会导致焦虑。

⑹．敌对

主要从三方面来反映敌对的表现：思想、感情及行为。其项目包括厌烦的感觉，摔物，争论直到不可控制的脾气暴发等各方面。

该分量表的得分在0-24分之间。得分在12分以上，表明个体易表现出敌对的思想、情感和行为。得分在6分以下表明个体容易表现出友好的思想、情感和行为。总的说来，得分越高，个体越容易敌对，好争论，脾气难以控制。得分越低，个体的脾气越温和，待人友好，不喜欢争论、无破坏行为。

⑺．恐怖

恐惧的对象包括出门旅行，空旷场地，人群或公共场所和交通工具。此外，还有社交恐怖。

该分量表的得分在0-28分之间。得分在14分以上，表明个体恐怖症状较为明显，常表现出社交、广场和人群恐惧，得分在7分以下，表明个体的恐怖症状不明显。总的说来，得分越高，个体越容易对一些场所和物体发生恐惧，并伴有明显的躯体症状。得分越低，个体越不易产生恐怖心理，越能正常的交往和活动。

⑻．偏执

主要指投射性思维，敌对，猜疑，妄想，被动体验和夸大等。

该分量表的得分在0-24分之间。得分在12分以上，表明个体的偏执症状明显，较易猜疑和敌对，得分在6分以下，表明个体的偏执症状不明显。总的说来，得分越高，个体越易偏执，表现出投射性的思维和妄想，得分越低，个体思维越不易走极端。

⑼．精神病性

反映各式各样的急性症状和行为，即限定不严的精神病性过程的症状表现。 　　该分量表的得分在0-40分之间。得分在20分以上，表明个体的精神病性症状较为明显，得分在10分以下，表明个体的精神病性症状不明显。总的说来，得分越高，越多的表现出精神病性症状和行为。得分越低，就越少表现出这些症状和行为。

（10）其它项目

作为附加项目或其他，作为第10个因子来处理，以便使各因子分之和等于总分。
