# Supplementary material for: Study of the mental health status of medical personnel dealing with new coronavirus pneumonia
Source: PLoS One. 2020 May 19;15(5):e0233145. doi: 10.1371/journal.pone.0233145 (PMC7237008; doi:10.1371/journal.pone.0233145)
Supplement: S2 Data — (DOC) [file pone.0233145.s004.doc]

1. Please ensure that the author list and affiliations are correct on the title page of your manuscript, and that your author contributions, competing interests, and financial disclosure are correct as listed below. All of these sections will be indexed in PubMed and published by PLOS ONE as you have written them. Please email plosone@plos.org if any changes to this content needs to be made.

ning sun

Conceptualization

Writing – original draft

Writing – review & editing

Jun Xing

Investigation

Methodology

Jun Xu

Resources

Validation

Writing – original draft

Shuling Geng

Data curation

Formal analysis

Yuqian Li

Data curation

Investigation

Writing – original draft

Please see here for the full list and definition of contributor roles: http://journals.plos.org/plosone/s/authorship#loc-author-contributions

Competing Interests: The authors have declared that no competing interests exist.

Financial Disclosure: The authors received no specific funding for this work

Answer: I’m sure that the author list and affiliations are correct on the title page of your manuscript,and that our author contributions, competing interests, and financial disclosure are correct as listed above. Competing Interests and Financial Disclosure were also right.

2. Please remove the Competing interests section and the author contributions from your manuscript as these will be published separately alongside your article.

Answer: We have removed the Competing interests section and the author contributions from our manuscript. Thanks

3. To prevent production delays, we recommend using the Author Formatting Checklist to confirm that your paper meets PLOS ONE's typesetting requirements for References, Tables, and Figures: http://journals.plos.org/plosone/s/file?id=c819/plos-one-author-formatting-checklist.docx.

This checklist is a reference tool for you; please do not upload the completed

Author Formatting Checklist with your submission files.

Answer: Thanks for your suggestion. We have used the Author Formatting Checklist to confirm that our paper meets PLOS ONE's typesetting requirements for References, Tables, and Figures.
