# Supplementary material for: Study of the mental health status of medical personnel dealing with new coronavirus pneumonia
Source: PLoS One. 2020 May 19;15(5):e0233145. doi: 10.1371/journal.pone.0233145 (PMC7237008; doi:10.1371/journal.pone.0233145)
Supplement: Attachment — Submitted filename: Response to Reviewers.doc [file pone.0233145.s005.doc]

Journal Requirements:

When submitting your revision, we need you to address these additional requirements:

1. Please ensure that your manuscript meets PLOS ONE's style requirements, including those for file naming. The PLOS ONE style templates can be found at http://www.plosone.org/attachments/PLOSOne_formatting_sample_main_body.pdf and <http://www.plosone.org/attachments/PLOSOne_formatting_sample_title_authors_affiliations.pdf>

Answer: We have checked the link and revise the manuscript according the PLOS ONE's style.

2. Please include in your Methods section (or in Supplementary Information files) the participating hospitals/institutions.

Answer: We have added the participating hospitals in the method section as following: The 12 hospitals including the 1^st^ Affiliated Hospital of Harbin Medical University, the 2^nd^ Affiliated Hospital of Harbin Medical University, Changchun Central Hospital, Liaoning Provincial People's Hospital, Qilu Hospital of Shandong University, the Second Hospital of Tianjin Medical University, People's Hospital of Inner Mongolia Autonomous Region, the First Hospital of Ningbo, the Second Hospital of Ningbo, Guizhou Provincial People's Hospital, Sichuan Provincial People's Hospital, and Wuhan University People's Hospital.

3. Please include additional information regarding the survey or questionnaire used in the study and ensure that you have provided sufficient details that others could replicate the analyses. If you developed and/or translated a questionnaire as part of this study and it is not under a copyright license more restrictive than Creative Commons Attribution (CC-BY), please include a copy, in both the original language and English, as Supporting Information.

Answer: we have added the details: It had been translated into Chinese version and used in the study. Both the original language and English version have been attached as supporting information.

4. We note that you have reported significance probabilities of 0 in places. Since p=0 is not strictly possible, please correct this to a more appropriate limit, eg 'p<0.0001'.

Answer: Thank you so much for your suggestion. We have use the p<0.0001 instead of the p=0

5. Thank you for stating the following financial disclosure:

"The funders had no role in study design, data collection and analysis, decision to publish, or preparation of the manuscript"

At this time, please address the following queries:

a. Please clarify the sources of funding (financial or material support) for your study. List the grants or organizations that supported your study, including funding received from your institution.
b. State what role the funders took in the study. If the funders had no role in your study, please state: “The funders had no role in study design, data collection and analysis, decision to publish, or preparation of the manuscript.”
c. If any authors received a salary from any of your funders, please state which authors and which funders.
d. If you did not receive any funding for this study, please state: “The authors received no specific funding for this work.”

Please include your amended statements within your cover letter; we will change the online submission form on your behalf.

Answer: Thank your so much for your suggestion. The study did not receive any funding. So we state: d “The authors received no specific funding for this work.” We have added the statement in our cover letter.

6. We note that you have indicated that data from this study are available upon request. PLOS only allows data to be available upon request if there are legal or ethical restrictions on sharing data publicly. For information on unacceptable data access restrictions, please see http://journals.plos.org/plosone/s/data-availability#loc-unacceptable-data-access-restrictions.

In your revised cover letter, please address the following prompts:

a) If there are ethical or legal restrictions on sharing a de-identified data set, please explain them in detail (e.g., data contain potentially identifying or sensitive patient information) and who has imposed them (e.g., an ethics committee). Please also provide contact information for a data access committee, ethics committee, or other institutional body to which data requests may be sent.

b) If there are no restrictions, please upload the minimal anonymized data set necessary to replicate your study findings as either Supporting Information files or to a stable, public repository and provide us with the relevant URLs, DOIs, or accession numbers. Please see http://www.bmj.com/content/340/bmj.c181.long for guidelines on how to de-identify and prepare clinical data for publication. For a list of acceptable repositories, please see http://journals.plos.org/plosone/s/data-availability#loc-recommended-repositories.

We will update your Data Availability statement on your behalf to reflect the information you provide.

Answer: Thank you so much for your direction. We have revised the content as following: All relevant data are within the manuscript and its Supporting Information files. And upload the data in the attachment. Thanks for your suggestion.

7. PLOS requires an ORCID iD for the corresponding author in Editorial Manager on papers submitted after December 6th, 2016. Please ensure that you have an ORCID iD and that it is validated in Editorial Manager. To do this, go to ‘Update my Information’ (in the upper left-hand corner of the main menu), and click on the Fetch/Validate link next to the ORCID field. This will take you to the ORCID site and allow you to create a new iD or authenticate a pre-existing iD in Editorial Manager. Please see the following video for instructions on linking an ORCID iD to your Editorial Manager account: <https://www.youtube.com/watch?v=_xcclfuvtxQ>

Answer: Thank you so much for your direction. I have had the ORCID ID in the past. When I validated it in Editorial Manager according the direction. The web showed the content as following:

But when I entered in the submit system. The web showed as following:

So if it have been validated or what can I do next? Thanks for your directing.

8. Your ethics statement must appear in the Methods section of your manuscript. If your ethics statement is written in any section besides the Methods, please move it to the Methods section and delete it from any other section. Please also ensure that your ethics statement is included in your manuscript, as the ethics section of your online submission will not be published alongside your manuscript.

Answer: I have deleted the ethics section after the discussion and put it in the Methods section according your request. Thank for your suggestion.

**Comments to the Author**
1. Is the manuscript technically sound, and do the data support the conclusions?
The manuscript must describe a technically sound piece of scientific research with data that supports the conclusions. Experiments must have been conducted rigorously, with appropriate controls, replication, and sample sizes. The conclusions must be drawn appropriately based on the data presented. 
Reviewer #1: Partly
Reviewer #2: Partly

Answer: We have read the content carefully. Thanks for your direction.

2. Has the statistical analysis been performed appropriately and rigorously?
   Reviewer #1: Yes
   Reviewer #2: Yes

Answer: We have read the content carefully. Thanks for your direction.

3. Have the authors made all data underlying the findings in their manuscript fully available?
   The [PLOS Data policy](http://www.plosone.org/static/policies.action#sharing) requires authors to make all data underlying the findings described in their manuscript fully available without restriction, with rare exception (please refer to the Data Availability Statement in the manuscript PDF file). The data should be provided as part of the manuscript or its supporting information, or deposited to a public repository. For example, in addition to summary statistics, the data points behind means, medians and variance measures should be available. If there are restrictions on publicly sharing data—e.g. participant privacy or use of data from a third party—those must be specified.
   Reviewer #1: Yes
   Reviewer #2: Yes

Answer: We have read the content carefully. Thanks for your direction.

4. Is the manuscript presented in an intelligible fashion and written in standard English?

   PLOS ONE does not copyedit accepted manuscripts, so the language in submitted articles must be clear, correct, and unambiguous. Any typographical or grammatical errors should be corrected at revision, so please note any specific errors here.
   Reviewer #1: Yes
   Reviewer #2: Yes

Answer: We have read the content carefully. Thanks for your direction.

5. Review Comments to the Author
   Please use the space provided to explain your answers to the questions above. You may also include additional comments for the author, including concerns about dual publication, research ethics, or publication ethics. (Please upload your review as an attachment if it exceeds 20,000 characters)
   Reviewer #1: The manuscript investigates an interesting topic that is becoming a challenging issue worldwide. The design limitations (lack of longitudinal design) could be tolerate due to the urge to disseminate research on the COVID19 pandemic. However, some modifications are requested to make the text more readable and some theoretical and background pitfalls should be addressed.

Answer: We have done some modifications making the text more readable and added some backgrounds.Thanks
The manuscript focuses on mental health status, and the main message is that mental health problems are a consequence of the emergency condition, and psychological strain linked to the fear of the disease plays a central role. However, more international references and literature on occupational stress, burnout and psychology of emergencies should be implemented in the introduction and in the discussion.

Answer: Thanks for your suggestion. We have added some references relating burnout and psychology of emergencies in the discussion.
Montemurro N. The emotional impact of COVID-19: from medical staff to common people.
Brain Behav Immun. 2020 Mar 30. pii: S0889-1591(20)30411-6. doi: 10.1016/j.bbi.2020.03.032. [Epub ahead of print]

Li Z, Ge J, Yang M et al.
Vicarious traumatization in the general public, members, and non-members of medical teams aiding in COVID-19 control.
Brain Behav Immun. 2020 Mar 10. pii: S0889-1591(20)30309-3. doi: 10.1016/j.bbi.2020.03.007. [Epub ahead of print]

Huang J, Liu F, Teng Z et al.
Care for the psychological status of frontline medical staff fighting against COVID-19.
Clin Infect Dis. 2020 Apr 3. pii: ciaa385. doi: 10.1093/cid/ciaa385. [Epub ahead of print]

[Goyal K](https://www.ncbi.nlm.nih.gov/pubmed/?term=Goyal K[Author]&cauthor=true&cauthor_uid=32143142), [Chauhan P](https://www.ncbi.nlm.nih.gov/pubmed/?term=Chauhan P[Author]&cauthor=true&cauthor_uid=32143142), [Chhikara K](https://www.ncbi.nlm.nih.gov/pubmed/?term=Chhikara K[Author]&cauthor=true&cauthor_uid=32143142), et al.Fear of COVID 2019: First suicidal case in India![J].[Asian J Psychiatr.](https://www.ncbi.nlm.nih.gov/pubmed/?term=Fear+of+COVID+2019:+First+suicidal+case+in+India!) Published online first 27 Feb 2020. DOI: 10.1016/j.ajp.2020.101989

Could you insert references of the Chinese adaptation of the SCL 90?

Answer: Thanks for your suggestion. We have inserted the reference of the Chinese adaptation of the SCL 90 as following: It had been translated into Chinese version and used in the study^[8]^.8.Wang YY, Jia XR, Song JQ, et al. Mental health status of medical staff during the outbreak of Coronavirus Disease 2019[J]. M edical Journal of W uhan University, Published online first 17 Mar 2020. DOI**:** 10. 14188/j. 1671⁃8852. 2020. 0154.
Please explain in the text what does the hospital class means.

Answer: Hospital class apply with the Chinese hierarchical hospital management. The management system divides hospitals into first, second and third levels. The division of grades is based on the number of beds: less than 100 beds are first-class hospitals; 101 beds to 500 beds are classified as second-level hospitals; More than five hundred, set as three level hospitals.

Could you provide in table 2 the number or the percentage of staff members who scored higher than the national norm cut off for each scl 90 subscale and for the total score? This would give the reader a synthetic idea of the mental health status of the personnel.

Answer: Thanks so much for your suggestion. We have added the number or the percentage of staff members who scored higher than the national norm cut off for each scl 90 subscale and for the total score in table2 according your suggestion making reader a synthetic idea of the mental health status of the personnel.

Regression analysis. Could you move the description of the stepwise model in the method paragraph, including table 3? You may keep the model results, including table 4, in the results section. This would make the results clearer and more readable.

Answer: We have delete the table3 according your suggestion making the results clearer and more readable.
Reviewer #2: Interesting paper. Research subjects and methods are correct.
Discussion must be improved. These papers can be intersting and related with your paper:
Montemurro N. The emotional impact of COVID-19: from medical staff to common people.
Brain Behav Immun. 2020 Mar 30. pii: S0889-1591(20)30411-6. doi: 10.1016/j.bbi.2020.03.032. [Epub ahead of print]

Li Z, Ge J, Yang M et al.
Vicarious traumatization in the general public, members, and non-members of medical teams aiding in COVID-19 control.
Brain Behav Immun. 2020 Mar 10. pii: S0889-1591(20)30309-3. doi: 10.1016/j.bbi.2020.03.007. [Epub ahead of print]

Huang J, Liu F, Teng Z et al.
Care for the psychological status of frontline medical staff fighting against COVID-19.
Clin Infect Dis. 2020 Apr 3. pii: ciaa385. doi: 10.1093/cid/ciaa385. [Epub ahead of print]

Answer: We have inserted the references in the discussion and reference. Thanks for your direction.

6.PLOS authors have the option to publish the peer review history of their article ([what does this mean?](https://journals.plos.org/plosone/s/editorial-and-peer-review-process#loc-peer-review-history)). If published, this will include your full peer review and any attached files.
If you choose “no”, your identity will remain anonymous but your review may still be made public.
**Do you want your identity to be public for this peer review?** For information about this choice, including consent withdrawal, please see our [Privacy Policy](https://www.plos.org/privacy-policy).
Reviewer #1: Yes: Filippo Rapisarda
Reviewer #2: No
[NOTE: If reviewer comments were submitted as an attachment file, they will be attached to this email and accessible via the submission site. Please log into your account, locate the manuscript record, and check for the action link "View Attachments". If this link does not appear, there are no attachment files to be viewed.]
While revising your submission, please upload your figure files to the Preflight Analysis and Conversion Engine (PACE) digital diagnostic tool, <https://pacev2.apexcovantage.com/>. PACE helps ensure that figures meet PLOS requirements. To use PACE, you must first register as a user. Registration is free. Then, login and navigate to the UPLOAD tab, where you will find detailed instructions on how to use the tool. If you encounter any issues or have any questions when using PACE, please email us at [figures@plos.org](mailto:figures@plos.org). Please note that Supporting Information files do not need this step.

Answer: We have read the content carefully. Thanks for your direction. The manuscript have no figures just tables.
